# Size-Dependent Cytotoxicity and Multi-Omic Changes Induced by Amorphous Silicon Nanoparticles in HepG2 Cells

**DOI:** 10.3390/toxics13040232

**Published:** 2025-03-21

**Authors:** Jiaqi Shi, Huifang Zhang, Yi Zhang, Ying Ma, Nairui Yu, Wenhao Liu, Ying Liu, Jisheng Nie, Zhangjian Chen, Guang Jia

**Affiliations:** 1 Department of Occupational and Environmental Health Sciences, School of Public Health, Peking University, Beijing 100191, China; 2311110200@bjmu.edu.cn (J.S.); 1710306142@pku.edu.cn (Y.Z.); mayingmmyy@163.com (Y.M.); 1810306112@bjmu.edu.cn (N.Y.); jiaguangjia@bjmu.edu.cn (G.J.); 2 Beijing Key Laboratory of Toxicological Research and Risk Assessment for Food Safety, School of Public Health, Peking University, Beijing 100191, China; 3 Shanxi Key Laboratory of Environmental Health Impairment and Prevention, NHC Key Laboratory of Pneumoconiosis, MOE Key Laboratory of Coal Environmental Pathogenicity and Prevention, School of Public Health, Shanxi Medical University, Taiyuan 030001, China; zhf201101@sxmu.edu.cn; 4 CAS Key Laboratory for Biomedical Effects of Nanomaterials and Nanosafety, CAS Center for Excellence in Nanoscience, National Center for Nanoscience and Technology, Beijing 100190, China; wenhaoliu2020@163.com (W.L.); liuy@nanoctr.cn (Y.L.)

**Keywords:** metabolomics, multi-omics, nanomaterials, proteomics, silica nanoparticles

## Abstract

(1) Background: Silica nanoparticles (SiO_2_ NPs) have a high potential for human exposure and tend to accumulate in the liver. This study aimed to explore the size-dependent cytotoxicity induced by SiO_2_ NPs and identify key molecular pathways at the in vitro level through proteomics, metabolomics, and a combination of multiple omics methods. (2) Methods: The human hepatoma cells (HepG2) cells were exposed to SiO_2_ NPs of three different sizes (60, 250, and 400 nm) at doses of 0, 12.5, 25, 50, 100, and 200 μg/mL for 24 h. (3) Results: Exposure to 60 nm SiO_2_ NPs induced more reduction in cell viability than the other two larger-scale particles. Changes in the metabolomic and proteomic profiles of HepG2 cells induced by SiO_2_ NPs were also size-dependent. The main pathways that were significantly affected in the 60 nm SiO_2_ NPs treatment group represented cholesterol metabolism in proteomics and central carbon metabolism in metabolomics. Moreover, common enrichment pathways between differential proteins and metabolites included protein digestion and absorption and vitamin digestion and absorption. (4) Conclusions: Exposure to SiO_2_ NPs could induce size-dependent cytotoxicity and changes in proteomics and metabolomics, probably mainly by interfering with energy metabolism pathways.

## 1. Introduction

Silica particles are abundant in nature and mainly in two forms: crystalline and amorphous. The chemical formula for crystalline silica and amorphous silica is the same, yet their structures differ. Crystalline silica is arranged regularly, while amorphous silica is arranged in disorder. Crystalline silica includes tridymite, quartz, and cristobalite. Amorphous silica includes mesoporous silica and crystalline silica. There are three sources of amorphous silica: natural existence, the by-product of power stations or metallurgical processing, and synthesis [1]. Silicon dioxide is one of the most produced nanomaterials in the world [2]. Synthetic amorphous silica (known as E551 in the European Union) is widely used in processed foods, for example, to prevent powdered products from clumping and for thickening paste products [3]. SiO_2_ NPs have some unique properties, such as larger specific surface area and pore volume, controllable particle size, easy-to-modify surfaces, excellent porosity, good biocompatibility, etc. Because of these characteristics, porous SiO_2_ NPs are widely used in drug delivery and biomedicine [4,5], coatings [6,7], cosmetics [8], etc. Therefore, SiO_2_ NPs have a high potential for human exposure through food, industrial, pharmaceutical, and cosmetic exposure, and they enter the body through oral, inhalation, intravenous, and skin-to-skin contact methods.

Many in vivo and in vitro experiments have shown that SiO_2_ NPs may can cause harm to a variety of organs, including the liver and spleen [9], lungs, immune system [10], cardiovascular system [11], and intestines [12]. Hepatotoxicity was one of the target-organ effects of oral exposure to nanomaterials, including SiO_2_ NPs [13,14,15]. Previous in vivo studies have found that SiO_2_ NPs tend to accumulate in the liver and cause pathological changes after oral exposure [16,17]. Furthermore, other investigations demonstrated the transport of SiO_2_ NPs to the liver in mice via intratracheal instillation [18] or intravenous exposure [9,19]. In several subchronic and chronic animal trials, SiO_2_ NPs were shown to generate adverse hepatotoxicity results such as liver atrophy, fatty liver, and hepatic fibrosis [20,21]. For example, adult male Sprague–Dawley rats treated with 1000 ppm SiO_2_ NPs for 28 days showed severe vacuolar degeneration of hepatocytes and focal coagulation necrosis of some hepatocytes [16]. In addition, smaller-sized SiO_2_ NPs can cause liver damage even at a lower dose [22,23]. In vitro experiments have also shown that SiO_2_ NPs can induce oxidative stress [24,25] and DNA damage [26] and increase mitochondrial-induced apoptosis [27] in human hepatoma cells. However, the key mechanism and toxic pathway of hepatotoxicity induced by SiO_2_ NPs as well as its size dependence are still unclear and need further study. The human hepatoma cells (HepG2) are widely used to study hepatotoxicity experimental materials [28,29,30,31,32]. Although HepG2 cells are a type of liver cancer cells, they can perform many differentiated liver functions and are often used as an in vitro substitute for primary human hepatocytes. Therefore, the cytotoxicity of SiO_2_ NPs of different sizes was carried out to provide ideas and clues for toxicity.

High-throughput technologies like proteomics and metabolomics have arisen and are still developing as a result of the ongoing advancements in biotechnology, which are aimed at determining the mechanism of toxicity [33,34,35]. Increasingly, in vitro cell studies and omics technologies are being used in nanotoxicology to investigate toxicity pathways and mechanisms [36,37,38]. Proteomics is the study of the whole complement of proteins in cells, their structure and function, and the identification of biomarkers that can forecast both qualitative and quantitative changes in cells upon exposure to harmful substances [39]. Compared to the transcriptome or proteome, metabolomics measures the total metabolites in living systems and concentrates on metabolic pathways, which are more representative of the cellular phenotype [40]. Multiple omics can integrate phenotypic changes at different levels to demonstrate a more comprehensive biological phenotype and better proof, whereas individual omics can indicate cellular phenotypic changes at separate molecular levels. The function of biomolecules can be thoroughly examined using multi-omics combination analysis, which can also screen important proteins, or metabolic pathways and provide coordination mechanisms for various biological layers.

Proteomics and metabolomics were employed in this study to investigate the fundamental toxic pathway and demonstrate the cytotoxicity of SiO_2_ NPs in human hepatoma cells (HepG2). In order to investigate the size dependency of the cytotoxicity, three amorphous SiO_2_ NPs with varying diameters (60, 250, and 400 nm) were chosen as the test nanoparticles. In order to properly screen for important proteins and metabolites linked to the liver cytotoxicity of SiO_2_ NPs and associated hazardous pathways, a correlation study between proteomics and metabolomics was also conducted.

## 2. Materials and Methods

### 2.1. Characterization of SiO_2_ NPs

Three different sizes (60, 250, and 400 nm) of silica nanoparticles (SiO_2_ NPs) were synthesized by the National Center for Nanoscience and Technology (Beijing, China). The particle size and dispersity were measured by transmission electron microscope (TEM; JEOL JEM-200CX, Tokyo, Japan) and quantitatively analyzed by GMS 3.0 software. To evaluate the agglomeration and stabilization of nanoparticles in ultrapure water, dynamic light scattering (DLS) was used to detect the hydrodynamic diameters and zeta potential of SiO_2_ NPs (at the concentration of 100 μg/mL) in ultrapure water using the ZetaSizer Nano ZS90 (Malvern Instruments Ltd., Malvern, UK).

### 2.2. Cell Culture and Exposure to SiO_2_ NPs

Human hepatocellular carcinoma cells (HepG2) used in the study were purchased from the National Biomedical Experimental Cell Resource Library of China. HepG2 cells were grown in Minimum Essential Medium (MEM, Gibco, New York, NY, USA) supplemented with 10% fetal bovine serum (FBS, Hyclone, Thermo Scientific, New York, NY, USA), 1% MEM Non-Essential Amino Acids Solution (100×) (NEAA, Gibco, Thermo Scientific, New York, NY, USA), and 2% GlutaMAX-1 (Gibco, Thermo Scientific, New York, NY, USA) and maintained at 37 °C in a humidified atmosphere containing 5% CO_2_. After reaching 80% confluence, the cells were digested by 0.25% trypsin and seeded to 96-well plates at a density of 1 × 10^4^ cells per well or 60 × 15 MM plates with 5 × 10^5^ cells per well. After a 36 h cultivation period, the medium was replaced by the fresh medium that contained different concentrations of SiO_2_ NPs suspensions.

### 2.3. Cell Viability Assay

To determine the cytotoxicity of SiO_2_ NPs, cell viability was assessed by the Cell Counting Kit-8 assay (CCK-8, Biotopped, Dojindo Laboratories, Kumamoto, Japan). After exposure to 0, 12.5, 25, 50, 100, and 200 μg/mL SiO_2_ NPs (60, 250 and 400 nm) for 24 h, the cells in the 96-well plate were incubated with CCK-8 solution for 2 h. Then, the supernatants were collected, and a microplate reader was used to detect the value of optical density (OD) at 450 nm. The cell viability was assessed by the following formula: [(experimental wells − blank wells)/(control wells − blank wells)] × 100%.

### 2.4. Proteomics

#### 2.4.1. Protein Sample Preparation

HepG2 (1 × 10^7^/sample) was exposed to SiO_2_ NPs (0 and 100 μg/mL) for 24 h and then lysed with lysate containing 1 mM PMSF. Three biological replicates were set up. Protein concentration was measured using the BCA assay kit (ThermoFisher, New York, NY, USA). Next, 50 μg of protein per sample was taken and different groups of samples diluted with lysate to the same concentration and volume. Then, 1/50 sample mass was added to 1 mg/mL pancreatic enzyme Trypsin-TPCK and digested overnight at 37 °C. After transferring the samples to a new tube, a labeling reaction was performed using a TMTpro reagent containing 20 μL of anhydrous acetonitrile (ThermoFisher, USA); then, it was incubated for 1 h at room temperature. The reaction was terminated with 5 μL 5% hydroxylamine for 15 min, and the aliquots were stored at −80 °C for later use.

#### 2.4.2. High-pH Reverse-Phase Separation and Mass Spectrometry (HPLC-MS) Assay

Reverse-phase separation was performed on an Agilent 1100 HPLC, and the gradient consisted of mobile phases A (H2O-FA (99.9:0.1, *v*/*v*)) and B (ACN-H2O-FA (80:19.9:0.1, *v*/*v*/*v*)). Samples were loaded at a flow rate of 2 μL/min to pre-column Acclaim PepMap100, 100 μm × 2 cm (RP-C18, ThermoFisher, New York, NY, USA), and then separated by Acclaim PepMap RSLC, 75 μm × 50 cm (RP-C18, ThermoFisher, New York, NY, USA), at the flow rate of 300 μL/ min. Samples were collected for 8–60 min. Samples were lyophilized for mass spectrometry (MS) using EASY-NLC 1200 liquid chromatography (ThermoFisher, New York, NY, USA). The mass range of the full MS scan was set at 350–1500 *m*/*z*, and the 20 highest peaks were MS/MS-scanned.

#### 2.4.3. Proteomic Data Analysis

All raw data were analyzed using Proteome Discover 2.4. Then, the trusted proteins were screened according to the Score Sequest HT > 0 and the unique peptide ≥ 1, and the blank values were removed. Differential proteins were screened on the condition that foldchange > 1.2 times and *p*-value < 0.05. The differential proteins were then analyzed for GO/KEGG enrichment to describe their functions.

### 2.5. Metabolomics

#### 2.5.1. Metabolomic Sample Preparation

HepG2 (1 × 10^7^/sample) was exposed to SiO_2_ NPs (0 and 100 μg/mL) for 24 h. The sample was treated with 20 μL of internal standard (L-2-chlorophenyl alanine, 0.06 mg/mL, methanol configuration), pre-chilled methanol/water (V:V = 4:1), and 200 μL of chloroform and then sonicated in the ice water bath for 20 min and centrifuged for 10 min (13,000 rpm, 4 °C). The supernatant was evaporated with a centrifugal concentration dryer, i.e., 80 μL of methoxyamine hydrochloride pyridine solution (15 mg/mL), and then vortexed for 2 min. An oxime reaction was performed in a 37 °C shaking incubator for 90 min. The sample was treated with 50 μL of BSTFA (containing 1% TMCS) derivatization reagent, 20 μL of n-hexane, and 10 μL internal standards (C8/C9/C10/C12/C14/C16/C18/ C20/C22/C24; all were configured with chloroform) at 70 °C for 60 min. At last, the sample was kept at room temperature for 30 min for GC-MS metabolomics analysis.

#### 2.5.2. Gas Chromatography–Mass Spectrometry (GC-MS) Analysis

Metabolic spectra were analyzed using gas chromatography–mass spectrometry (7890B-5977A, Agilent, Santa Clara, CA, USA). DB-5MS capillary columns (30 m × 0.25 mm × 0.25 μm, Agilent J&W Scientific, Folsom, CA, USA) were used; the carrier gas was high-purity helium (purity was not less than 99.999%), the flow rate was 1.0 mL/min, and the temperature of the inlet was 260 °C. Electron bombardment of the ion source (EI) was used at a temperature of 230 °C, with a four-stage rod temperature of 150 °C and electron energy of 70 eV. The scanning mode was the full-scan mode (SCAN), and the quality scanning range was 50–500 *m*/*z*.

#### 2.5.3. Metabolomic Data Analysis

Qualitative and relative quantitative analyses of the original non-targeted metabolomics data were conducted by the software of MS-DIAL 4.70. After standardization of the original data, unsupervised principal component analysis (PCA) and supervised orthogonal partial least squares analysis (OPLS-DA) were used to observe the overall distribution. The criteria for screening were the VIP value of the first principal component of the OPLS-DA model > 1 and the *p*-value value of the *t*-test < 0.05. Finally, based on the KEGG database, the differential metabolites were enriched by metabolic pathway enrichment.

### 2.6. Integrative Metabolomics and Proteomics Analysis

To determine their shared pathway information, the differential proteins and differential metabolites were concurrently mapped to the KEGG pathway database using the MetaboAnalyst 6.0 platform. The linkages between graphical objects in the KEGG pathway and details about the orthogenic genes in the KEGG GENES database are both contained in the KGML (KEGG Markup Language) file, a sub-library of the KEGG database. A more methodical investigation of the relationship between the proteome and the metabolome is made possible by the ability to determine the network relationship between proteins and metabolites.

### 2.7. Statistical Analysis

For statistical analysis, R software (version 4.1.1) was utilized. Additionally, all of the outcomes were mean ± SD. When necessary, one-way ANOVA with Tukey’s correction and unpaired Student’s *t*-test were employed. For all data, a *p*-value of less than 0.05 was deemed statistically significant.

## 3. Results

### 3.1. Characterization of SiO_2_ NPs

**Figure 1:**
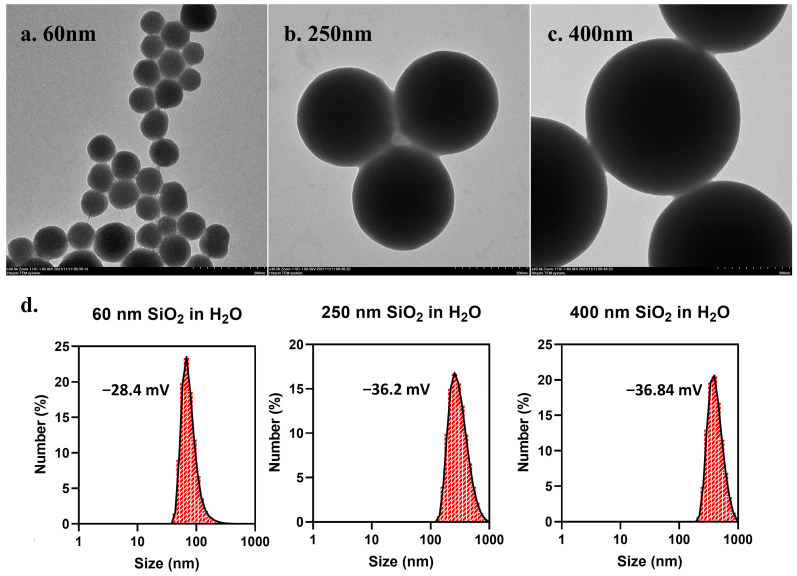
**Characterization of SiO_2_ NPs.** The morphologies of SiO_2_ NPs at 60 nm (**a**), 250 nm (**b**), and 400 nm (**c**) were observed under transmission electron microscopy (TEM). The hydrated particle size distribution and zeta potential of SiO_2_ NPs of three different sizes in ultrapure water were detected by DLS (**d**).

SiO_2_ NPs were characterized before use. Transmission electron microscopy (TEM) images showed that the 60 nm, 250 nm, and 400 nm SiO_2_ NPs used in this study were all spherical and well dispersed (Figure 1a–c), with equivalent diameters of 73 ± 3.573 nm, 278.41 ± 10.324 nm, and 426.173 ± 27.428 nm, respectively. The hydrated particle sizes of these three SiO_2_ NPs in ultrapure water were 173.8 ± 6.259 nm, 472.4 ± 14.81 nm, and 500.7 ± 14.16 nm, respectively, and zeta potentials were −28.4 ± 0.424 mV, −36.2 ± 0.696 mV, and −36.84 ± 0.865 mV, respectively. This indicates that SiO_2_ NPs still had slight agglomeration in an aqueous solution, even if they were well dispersed.

### 3.2. Cytotoxicity of SiO_2_ NPs in HepG2 Cells

**Figure 2:**
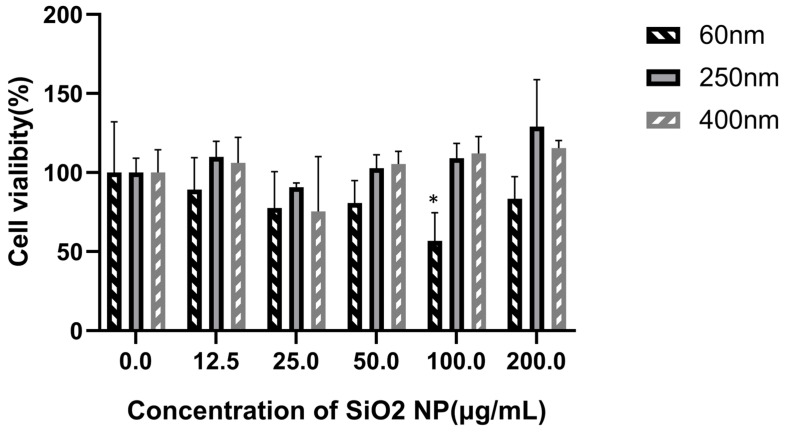
**Effect of SiO_2_ NPs on the viability of HepG2 cells (mean ± SD, *n* = 3).** HepG2 cells were treated with SiO_2_ NPs (60, 250, and 400 nm) at 0, 12.5, 25, 50, 100, and 200 μg/mL for 24 h. The cell viability was significantly decreased in the 60 nm SiO_2_ NPs treatment groups when the treatment concentration was 100 μg/mL, but no obvious change was found in the 250 nm and 400 nm SiO_2_ NPs treatment groups. Cell viability did not decrease in a dose-dependent relationship. Significant difference from the control (* *p* < 0.05).

The cell viability was measured using the CCK-8 kit after treatment of SiO_2_ NPs with three different sizes at gradient concentrations for 24 h. As shown in Figure 2, cell viability was decreased after exposure to SiO_2_ NPs, and 60 nm SiO_2_ NPs induced more reduction in cell viability than the other two larger-scale particles. Compared with the control group (0 μg/mL), the 60 nm SiO_2_ NPs treatment group at a dose of 100 μg/mL reduced the cell viability significantly. However, the result did not show a significant dose–response relationship. After exposure to 60 nm SiO_2_ NPs, cell viability in the 12.5, 25, 50, 100, and 200 μg/mL groups was reduced to 89.30%, 77.56%, 76.11%, 80.81%, and 83.49%, respectively. Cell viability after exposure to the other two larger-scale SiO_2_ NPs (250 and 400 nm) at these doses barely showed any cytotoxicity.

### 3.3. Proteomics

**Figure 3:**
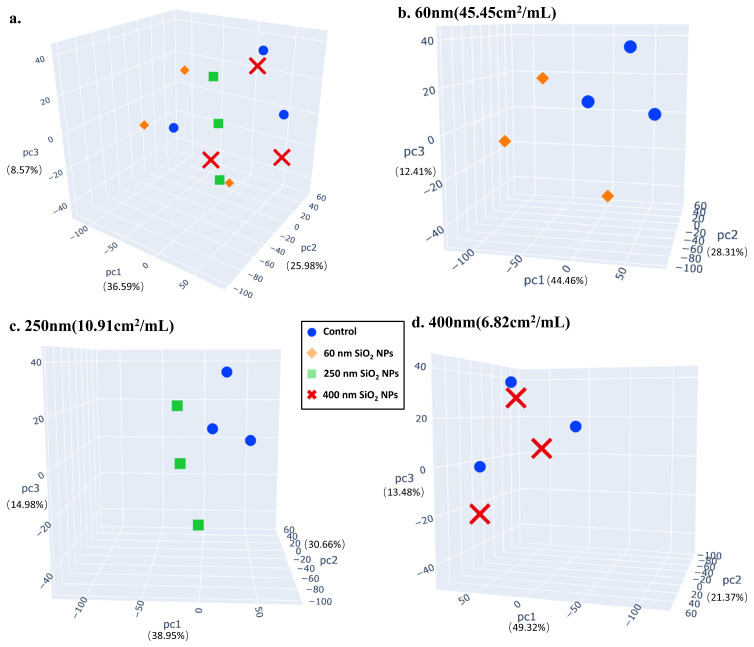
**Multivariate analysis of proteomics in the control group and SiO_2_ NPs-treated (100 μg/mL) groups.** PCA 3D plots were drawn to compare the difference between the control group and the treatment groups (**a**). Pairwise comparisons between the control group and 60 nm (**b**), 250 nm (**c**), and 400 nm (**d**) treatment groups were also carried out. The control group and the three SiO_2_ NPs treatment groups were relatively far apart on the score map, revealing significantly separations, especially between the 60 nm or 250 nm SiO_2_ NPs treatment group and the control group (control-1, control-2, and control-3).

After being treated with 100 μg/mL SiO_2_ NPs for 24 h, HepG2 cells were lysed, and proteins were extracted. Proteomics was performed in HPLC-MS. Through the processing of raw MS data, 67,388 peptides were extracted, and 6829 protein groups were identified. As shown in Figure 3, PCA scoring plots revealed a significant difference between the control group and the three SiO_2_ NPs treatment groups, especially between the 60 nm or 250 nm SiO_2_ NPs treatment group and control group, in which the difference was shown in proteomics characteristics.

**Figure 4:**
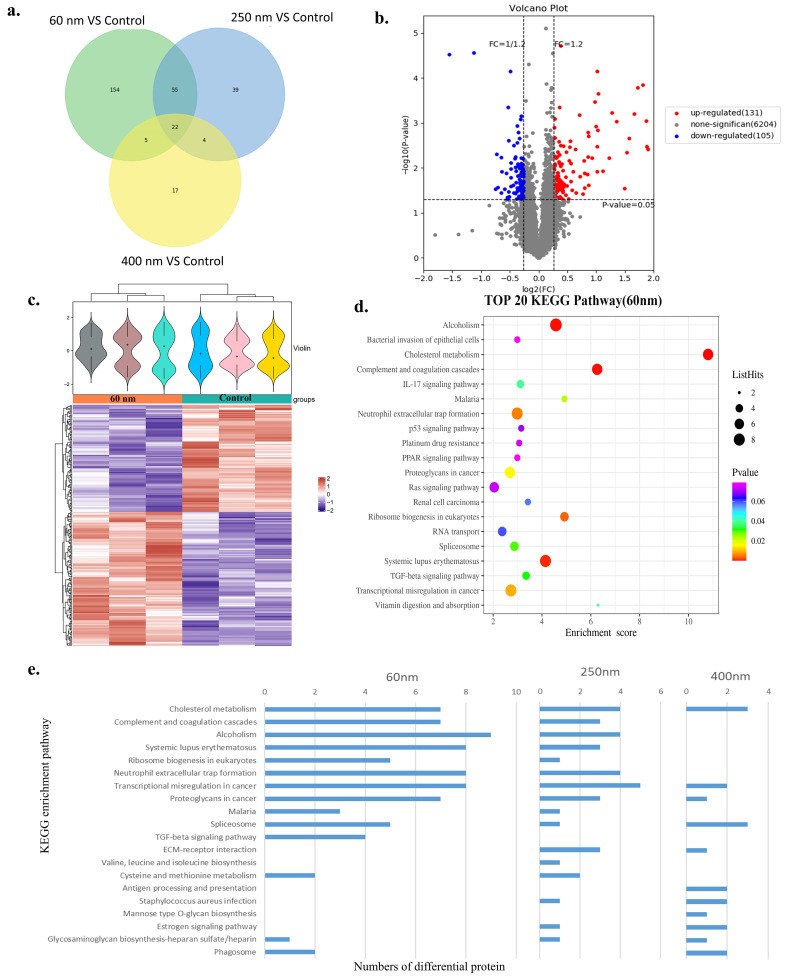
**Proteomics and pathway analysis of SiO_2_ NPs.** A Venn diagram of the different proteins in three treatment groups compared to the control group showing that the size of SiO_2_ NPs affected different proteins (**a**). A volcano map of proteins with varying expression levels in the 60 nm treatment group (**b**). A heat map of cluster analysis comparing the 60 nm treatment and control groups revealing a notable difference (**c**). The KEGG enrichment analysis bubble plot, generated in descending order of −log10 *p*-value in the 60 nm SiO_2_ NPs treatment group (**d**). The bar pattern of different protein numbers in different pathways of KEGG compares the three particle-size treatment groups (**e**).

**Figure A1:**
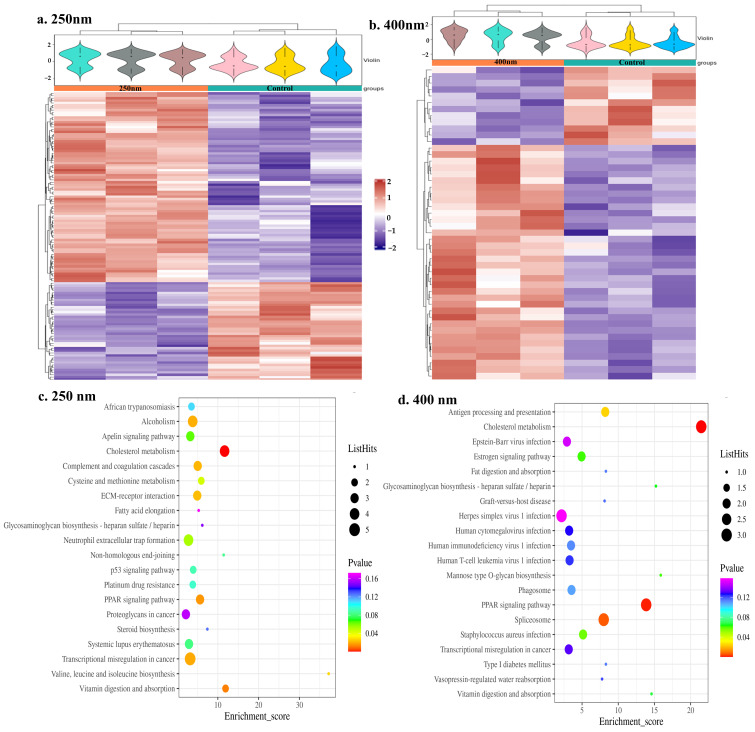
**Proteomics and pathway analysis in 250 nm and 400 nm SiO_2_ NPs treatment groups.** The heat map of cluster analysis demonstrates the characteristic difference between 250 nm (**a**) or 400 nm (**b**) treatment group and control group. KEGG enrichment analysis bubble plots drawn in the descending order of −log1010 *p*-value corresponding to each entry in 250 nm SiO_2_ NPs treatment group (**c**) and 400 nm SiO_2_ NPs treatment group (**d**).

Then, differentially expressed proteins between the SiO_2_ NPs treatment groups and control groups were screened. It was found that there were 236, 120, and 48 differential proteins in the 60 nm, 250 nm, and 400 nm SiO_2_ NPs treatment groups, respectively (Figure 4a). This suggests that the smaller the particle size of SiO_2_ NPs, the more differential proteins affected, and the greater the effect on cells. Meanwhile, these differential proteins intersected with other size groups, but the proportion was less than 50%, which meant that SiO2 particles as small as 60 nm may induce many new effects on cell protein expression. Among 236 differential proteins between the 60 nm SiO_2_ NPs treatment group and control group, 131 proteins were up-regulated, and 105 were down-regulated (Figure 4b). The cluster heat map of these differential proteins visually demonstrates the characteristic difference between SiO_2_ NPs treatment groups and control groups (Figure 4c and Figure A1a,b).

Based on KEGG enrichment pathway analysis (Figure 4d,e and Figure A1c,d), the main pathways that were significantly affected in the 60 nm SiO_2_ NPs treatment group included cholesterol metabolism, complement and coagulation cascades, alcoholism, etc. Compared with the 250 nm and 400 nm SiO_2_ NPs, 60 nm SiO_2_ NPs significantly enhanced pathways such as the Ras signaling pathway and weakened the pathways such as glycosaminoglycan biosynthesis–heparin sulfate/heparin. Therefore, the treatment of SiO_2_ NPs could induce size-dependent proteomic changes: the smaller the particle size, the greater the impact.

### 3.4. Metabolomics

**Figure 5:**
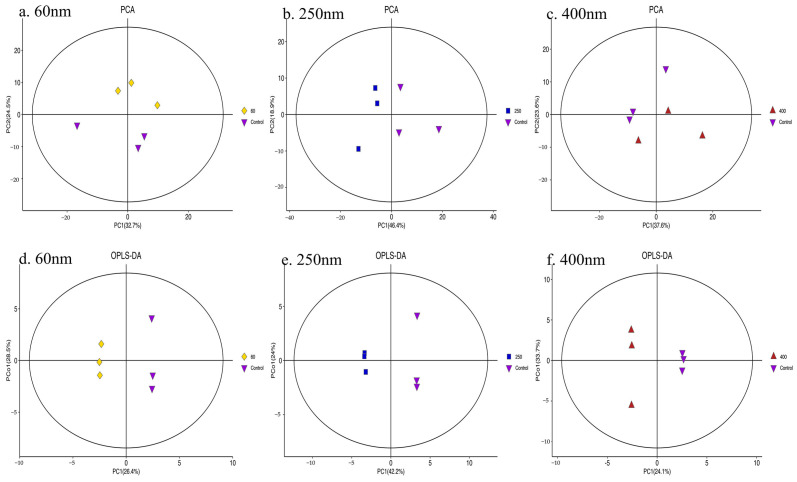
**Multivariate analysis of metabolites in the control group and SiO_2_ NPs-treated (100 μg/mL) groups.** Pairwise comparisons between the control group and 60 nm (**a**), 250 nm (**b**), and 400 nm (**c**) treatment groups carried out through PCA 2D plots. PCA was performed by the expression of trusted metabolites. The control group and the SiO_2_ NPs treatment groups of three sizes are significantly separated, indicating the difference in metabolic characteristics. OPLS-DA compares the difference between the control group and the treatment groups of three different sizes, respectively (**d**–**f**).

After being treated with 100 μg/mL SiO_2_ NPs for 24 h, HepG2 cells were also digested for untargeted metabolomics in GC-MS. After metabolite identification, 496 metabolites were included in subsequent analysis. As shown in Figure 5, PCA and OPLS-DA plots revealed that the control group and the three SiO_2_ NPs treatment groups were significantly separated, indicating a difference in metabolic characteristics.

**Figure 6:**
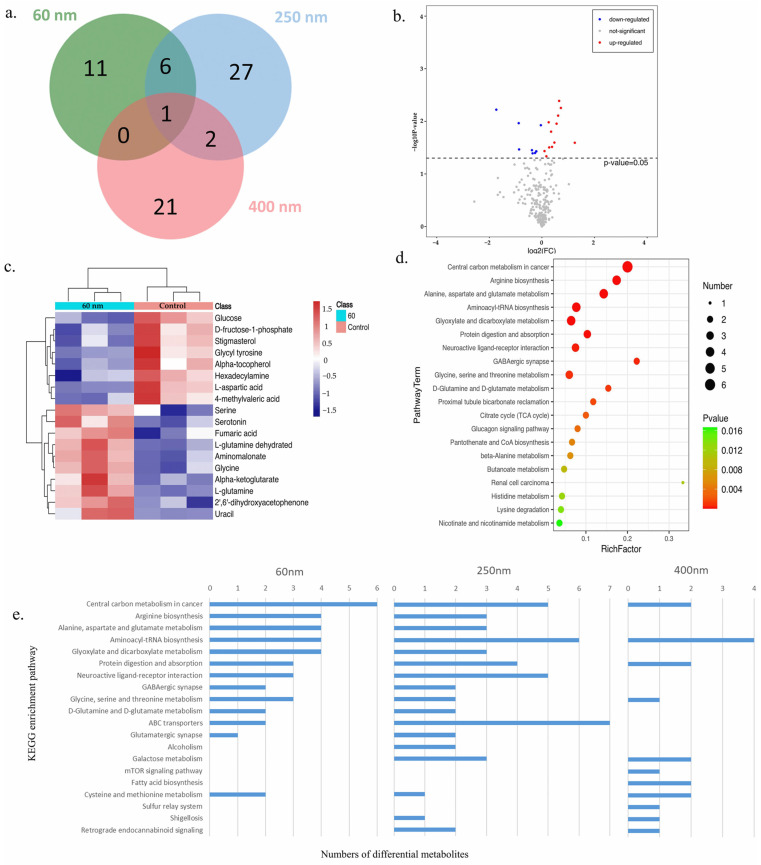
**Metabolomics and pathway analysis of HepG2 cells treated with SiO_2_ NPs.** A Venn diagram of the different metabolites in treatment groups of three different sizes compared with the control group demonstrates the difference in the size of SiO_2_ NPs on differential proteins (**a**). Differentially expressed metabolites in the 60 nm treatment group shown on a volcano map (**b**). The characteristic difference between the 60 nm treatment group and control group shown by a cluster analysis heat map (**c**). A KEGG enrichment analysis bubble plot drawn in the descending order of −log10 *p*-value corresponding to each entry in the 60 nm SiO_2_ NPs treatment group (**d**). The bar graph of different metabolite numbers in different pathways of KEGG comparing the three particle size treatment groups (**e**).

**Figure A2:**
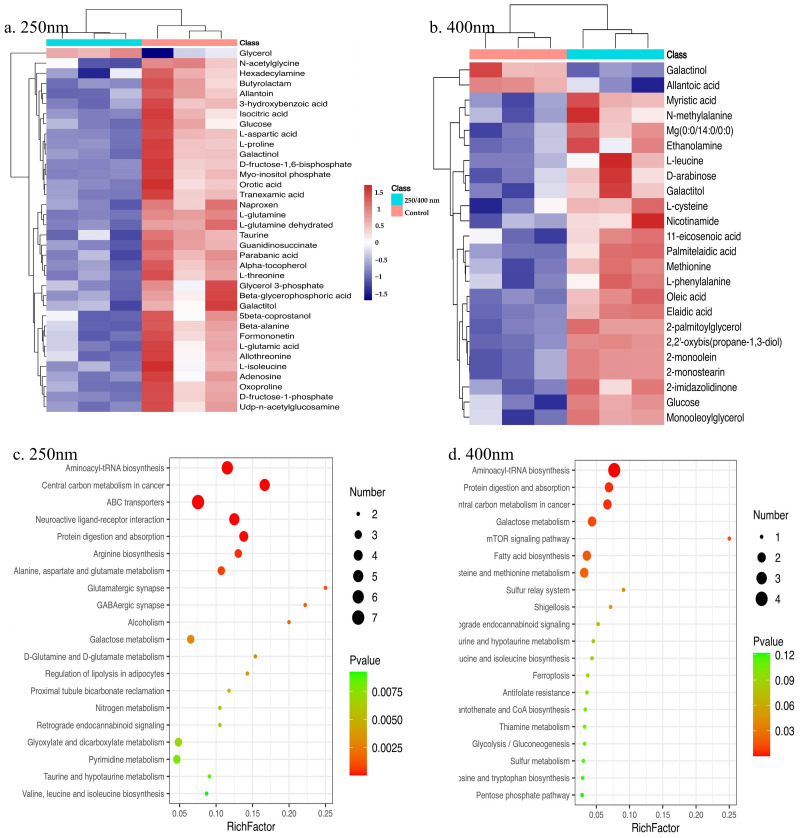
**Metabolomics and pathway analysis in 250 nm and 400 nm SiO_2_ NPs treatment groups.** The heat map of cluster analysis demonstrates the characteristic difference between 250 nm (**a**) or 400 nm (**b**) treatment group and control group. KEGG enrichment analysis bubble plots drawn in the descending order of −log1010 *p*-value corresponding to each entry in 250 nm (**c**) and 400 nm SiO_2_ NPs treatment group (**d**).

Then, differentially expressed metabolites between the SiO_2_ NPs treatment groups and control group were screened. It was found that there were 18, 36, and 24 differential metabolites in the 60 nm, 250 nm, and 400 nm SiO_2_ NPs treatment groups, respectively (Figure 6a). Among 18 differential metabolites between the 60 nm SiO_2_ NPs treatment group and control group, which were mainly divided into the category of lipids and lipid-like molecules, 8 proteins were up-regulated, and 10 were down-regulated (Figure 6b). However, most of the differential metabolites (97%) in the 250 nm SiO_2_ NPs treatment group were down-regulated, while most of the differential metabolites (92%) in the 400 nm SiO_2_ NPs treatment group were up-regulated. These results also indicate that SiO_2_ NPs with different particle sizes induced different metabolic changes. The cluster heat maps of the differential metabolites in the 60 nm, 250 nm, and 400 nm SiO_2_ NPs treatment groups compared with the control group are shown in Figure 6c and Figure A2a,b.

Based on KEGG enrichment pathway analysis (Figure 6d,e and Figure A2c,d), the main metabolic pathways that were significantly affected in the 60 nm SiO_2_ NPs treatment group included the central carbon metabolism in cancer, arginine biosynthesis, and alanine, aspartate, and glutamate metabolism pathways, among others. Compared with the 250 nm and 400 nm SiO_2_ NPs, 60 nm SiO_2_ NPs significantly enhanced pathways such as glyoxylate and dicarboxylate metabolism. Therefore, the treatment of SiO_2_ NPs could induce metabolic changes, which were shown to vary with the change in particle size.

### 3.5. Correlation Analysis of Proteomics and Metabolomics

**Figure 7:**
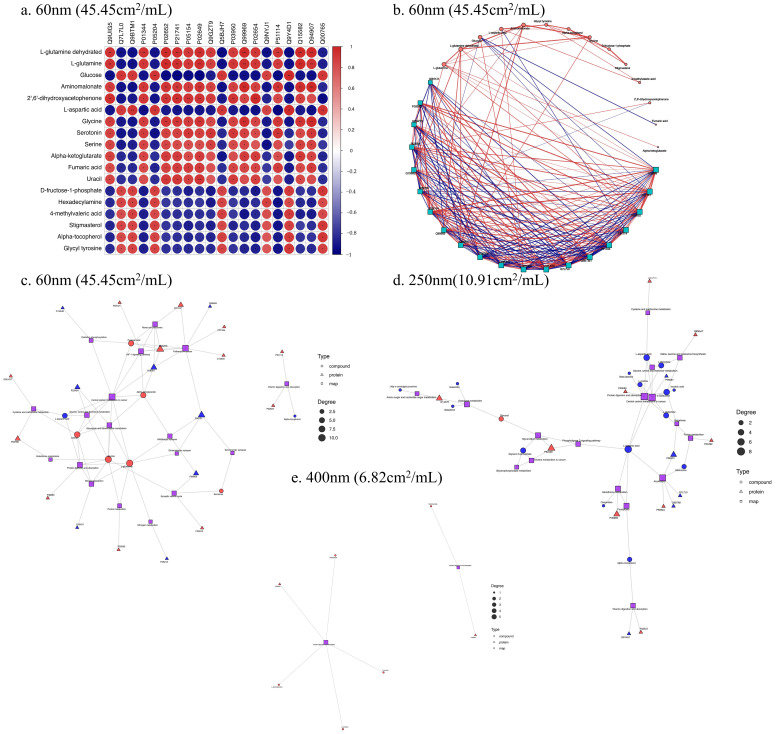
**Correlation analysis of proteomics and metabolomics.** A correlation heat map of differential proteins and differential metabolites treated with 60 nm SiO_2_ NPs (**a**) (* *p* < 0.05; ** *p* < 0.01, *** *p* < 0.001). The correlation network diagram drawn according to the correlation analysis results of the differential gene and the differential metabolite (**b**). In the figure, the shape size is related to the number of connections, and the thickness of the connection between the shapes represents the degree of correlation. In the KGML network diagrams of differential proteins and metabolites in the 60 nm (**c**), 250 nm (**d**), and 400 nm (**e**) SiO_2_ NPs treatment groups, the red color indicates up-regulated proteins or metabolites, and the blue color indicates down-regulated proteins or metabolites.

**Figure A3:**
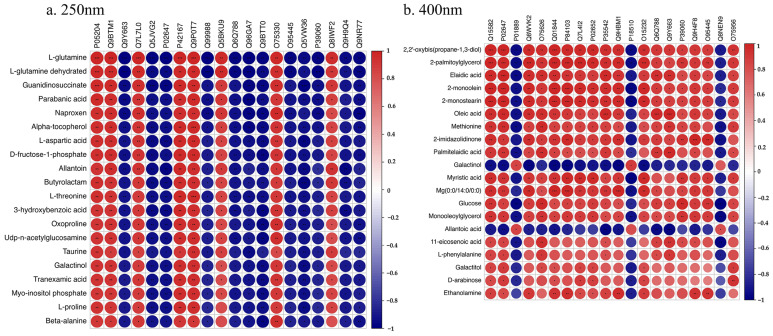
**Correlation heat map of differential proteins and differential metabolites treated with 250 nm (a) or 400 nm (b) SiO_2_ NPs** (* *p* < 0.05; ** *p* < 0.01; *** *p* < 0.001).

We further combined the results of proteomics and metabolomics to find key events at different molecular levels induced by SiO_2_ NPs. At first, the correlations between differential proteins and differential metabolites were confirmed by Pearson correlation analysis (Figure 7a and Figure A3). For example, among these differential proteins and metabolites induced by 60 nm SiO_2_ NPs, more than 86.9% were significantly correlated. According to the results of the correlation analysis between differential proteins and differential metabolites, the correlation network diagram was plotted (Figure 7b). The differential proteins apolipoprotein A-II (APOA2), midkine (MDK), and apolipoprotein E (APOE) and the differential metabolites L-glutamine dehydrated and L-glutamine were found to have the largest numbers of associations, respectively. Moreover, pathways in cancer and central carbon metabolism in cancer were the two pathways that had the largest numbers of associations in the 60 nm SiO_2_ NPs treatment group (Figure 7c), differing from the other two treatment groups (Figure 7d,e).

Through the analysis of the combined pathways of protein metabolism, it was found that the significantly enriched pathways were mainly as follows: arginine biosynthesis, complement and coagulation cascades, and cholesterol metabolism in the 60 nm group; galactose metabolism and valine, leucine, and isoleucine biosynthesis in the 250 nm group; and aminoacyl-tRNA biosynthesis in the 400 nm group. The two pathways cholesterol metabolism and central carbon metabolism in cancer were the common pathways between the three size groups. The pathway results of the 250 nm group and the 400 nm group were similar. Moreover, several key metabolic pathways were also screened by KGML analysis, including glyoxylate and dicarboxylate metabolism and amino acid metabolism (glycine, serine, and threonine metabolism). In addition, there were complex interactions between differential proteins (EGLN1 (Egl nine homolog 1) and MET (hepatocyte growth factor receptor)) and metabolites (fumaric acid and alpha-ketoglutarate), which can lead to SiO_2_ NPs-induced metabolic dysfunction.

## 4. Discussion

Humans experience exposure to SiO_2_ NPs through various pathways, and many reports are confirming the adverse effects of SiO_2_ NPs on human health. Therefore, studying the cytotoxic effects and mechanisms of SiO_2_ NPs is necessary for safety assessment. The liver is the main target organ for SiO_2_ NPs. The objective of this study was to analyze the effects of SiO_2_ NPs exposure on cellular metabolism based on proteomics and metabolomics techniques and to explore the mechanism of cytotoxicity. Through the differential proteins and differential metabolites screened after exposure to smaller-sized SiO_2_ NPs at 100 μg/mL, we found that SiO_2_ NPs could interfere with cholesterol metabolism, glucose metabolism, and amino acid metabolism, eventually leading to cytotoxicity. This provides clues for the potential mechanism underlying the cytotoxicity induced by SiO_2_ NPs. This study also helps to deepen our understanding of the potential health risks caused by SiO_2_ NPs and contributes to the application of combined omics in toxicological assessment. The harmful effects of silica are influenced by the size of the nanoparticles. The 60 nm treatment group in our study exhibited more cytotoxicity than the 250 nm and 400 nm groups. Numerous further in vitro and in vivo investigations have also demonstrated a strong correlation between the size of the particles and the cytotoxicity of SiO_2_ NPs [41,42]. Yang et al. studied the effects of four different-sized particles (68, 43, 19, and 498 nm) on HepG2 cells and found that smaller silica particles had higher toxic effects for the probable reason that smaller particles on the nanoscale were more easily endocytosed [23]. Demir et al. [43] used *Drosophila melanogaster* to detect the genotoxic activity of different SiO_2_ NPs with different sizes (6, 15, 30, and 55 nm) and observed significant induction of oxidative DNA damage, which was indirectly related to SiO_2_ NPs size. In addition, this study found that the omics results of SiO_2_ NPs are also highly correlated with the size of the particles, consistent with some other findings [44,45]. In proteomics, we found the particle size became smaller, and the number of differential proteins also became smaller (whether the differential proteins were up- or down-regulated). Similarly, Bannuscher et al. [44] treated NR8383 alveolar macrophages with three sizes of SiO_2_ NPs (7, 15, and 40 nm) and found that the overall clustering structure of the untargeted proteomics results was similar, but the counts of proteins decreased with size. Karkossa et al. [45] found similar results in that the amount of change in protein and metabolomics results was related to the size of the particles. Thus, consistent with previous findings, smaller particles tend to have higher cytotoxicity and bring greater perturbations at the protein or metabolic level than larger ones. At present, nanotoxicology studies are still basically based on mass concentration as the dose unit, and this study is the same. However, the toxicity of nanoparticles is affected by various physicochemical parameters, including size, surface structure, etc., and some studies have found that surface area is more effective as a dose index when studying particles of different size ranges [46,47], which is a follow-up aspect worthy of attention.

Proteomics is essential for understanding complex biochemical processes at the protein level. The results showed that protein synthesis-related pathways were affected by SiO_2_ NP, including ribosome, translational, and transcriptional pathways. In our study, 60 nm particles disrupted RNA modifications (NOP10, NHP2, and SNU13 down-regulated), splisomes (HNRNPM, RBMX, and SNU13 down-regulated), and ribosome proteins (MRPL11, MRPL14, and MRPL23 down-regulated). In addition, from the proteomics results, we found that SiO_2_ NP exposure also affected cholesterol metabolism, and the differential proteins related to cholesterol metabolism screened in this study were up-regulated, such as APOB, APOE, APOA1, APOA2, APOC1, ANGPTL8, and LRP2. Some studies have yielded the same or similar results. Duan et al. [48] used an ICR mouse model to find that the level of cholesterol and LDL cholesterol in serum and liver tissue was significantly increased, and the ratio of HDL to LDL cholesterol was significantly reduced A zebrafish biological model was used to find that SiO_2_ NP could activate lipid metabolism pathways. Chatterjee et al. [49] treated HepG2 with four types of amorphous SiO_2_ NPs with different surface areas and also found perturbations of steroid-cholesterol biosynthesis. This suggests that repeated exposure to SiO_2_ NP may be a risk factor for metabolic and cardiovascular diseases such as metabolic syndrome, non-alcoholic fatty liver disease, atherosclerosis, and type II diabetes [50].

SiO_2_ NPs interfered with several key metabolic pathways, including glyoxylate and dicarboxylate metabolism and glycine, serine, and threonine metabolism, which could exacerbate oxidative stress and lead to liver damage. These results are similar to those of other studies [48,51]. Chatterjee et al. [52] conducted metabolomics with amorphous SiO_2_ NP-treated HepG2 cells and ICR mouse livers and found that inhibition of glutathione metabolism and oxidative stress were some of the main causes of amorphous SiO_2_ NPs-mediated hepatotoxicity. Enrichment analysis of differential metabolites showed that arginine biosynthesis and alanine, aspartic acid, and glutamate metabolism were affected by SiO_2_ NP exposure. The liver is essential for maintaining normal glucose homeostasis, which ensures the energy supply of various tissues in the body through gluconeogenesis, glycogen decomposition, glycogen synthesis, glycolysis, and so on [53]. From the metabolomics results, we found that the differential metabolite (D-fructose-1-phosphate, beta-D-glucose) content associated with glucose metabolism decreased, and the glycolysis/gluconeogenesis pathway was blocked.

Through the joint pathway enrichment analysis of proteomics and metabolomics, it was also found that arginine biosynthesis and cholesterol metabolism were two significantly altered pathways, echoing the results of the above-mentioned single omics results. The correlation analysis showed that amino acid metabolism was also affected by SiO_2_ NP. In addition to being precursors to numerous metabolic intermediates, amino acids are essential for energy metabolism and protein synthesis. We found the content of α-ketoglutaric acid, fumaric acid, glycine, L-glutamine, and serine increased and the aspartic acid content decreased. Fumarate and α-ketoglutaric acid, the intermediates in the citric acid cycle (TCA), promote the accumulation of fumarate and succinate, respectively [54], diffusing in the cytosol and ultimately promoting a pseudo-hypoxic state that favors tumor development [55]. The three enzymes engaged in the de novo synthesis of purine nucleotides and the two enzymes involved in the de novo synthesis of pyrimidine nucleotides both require glutamine as a substrate [56]. In addition to the fact that a large part of the lactic acid produced in cancer cells comes from glutamine [57], glutamine decomposition and reducing carboxylation can lead to anabolic reactions of fatty acids and cholesterol. The one-carbon route, which involves serine and glycine, is involved in the production of purines and pyrimidines as well as the regulation of cancer cells’ epigenetic traits [58,59]. Aspartic acid is the only amino acid that can directly enter gluconeogenesis without entering the TCA cycle [60]; it is a carbon source synthesized by purine and pyrimidine, and the decrease in its expression directly affects the level of purine and pyrimidine [60]. Our results show that SiO_2_ NPs disrupt energy metabolism in HepG2 cells, similar to the results of previous studies [61].

The advantage of this study is its use of a combination of proteomics and metabolomics analysis, which helped to determine the functional pathways involved in cytotoxicity and elucidate its underlying mechanisms. Systems biology, including proteomics and metabolomics, provides a lot of data that can be used to reveal novel biomarkers and biological pathways involved in the way nanomaterials act. However, the wide range of physicochemical properties of proteins dictates that only a subset of all proteins are detected by current technology, and metabolomics data also show lower sensitivity than commonly used toxicological methods [62]. Therefore, by integrating results from multiple cellular reaction layers from different omics methods, a higher level of confidence can be obtained.

## 5. Conclusions

In summary, the present study focused on cytotoxicity and omics changes in HepG2cells after exposure to SiO_2_ NPs of different sizes. It was demonstrated that exposure to SiO_2_ NPs could induce size-dependent cytotoxicity and changes in proteomics and metabolomics, probably mainly by interfering with energy metabolism pathways, including cholesterol metabolism and arginine biosynthesis. Our findings provide scientific insights for the exploration of toxicity mechanisms of SiO_2_ NPs.

## Data Availability

Data are contained within the article and Appendix A.

## Abbreviations

The following abbreviations are used in this manuscript:

SiO_2_ NPs	Silica nanoparticles
HepG2	Human hepatoma cells
WHO	World Health Organization
CCK-8	Cell Counting Kit-8 assay
OD	Optical density
HPLC-MS	High-pH reverse-phase separation and mass spectrometry
GC-MS	Gas chromatography–mass spectrometry
PCA	Principal component analysis
OPLS-DA	Orthogonal partial least squares analysis
KGML	KEGG markup language
SD	Standard deviation
TEM	Transmission electron microscopy
APOA2	Apolipoprotein A-II
MDK	Midkine
APOE	Apolipoprotein E
EGLN1	Egl nine homolog 1
